# Perturbative method for maximum likelihood estimation of the Weibull distribution parameters

**DOI:** 10.1186/s40064-016-3500-y

**Published:** 2016-10-18

**Authors:** V. H. Coria, S. Maximov, F. Rivas-Dávalos, C. L. Melchor-Hernández

**Affiliations:** Instituto Tecnológico de Morelia, Morelia, México

**Keywords:** Weibull distribution, Maximum likelihood estimation, Parameter estimation, Censored data, Perturbation theory

## Abstract

The two-parameter Weibull distribution is the predominant distribution in reliability and lifetime data analysis. The classical approach for estimating the scale $(\alpha )$ and shape $(\beta )$ parameters employs the maximum likelihood estimation (MLE) method. However, most MLE based-methods resort to numerical or graphical techniques due to the lack of closed-form expressions for the Weibull $\beta$ parameter. A Weibull $\beta$ parameter estimator based on perturbation theory is proposed in this work. An explicit expression for $\beta$ is obtained, making the estimation of both parameters straightforward. Several right-censored lifetime data sets with different sample sizes and censoring percentages were analyzed in order to assess the performance of the proposed estimator. Study case results show that our parameter estimator provides solutions of high accuracy, overpassing limitations of other parameter estimators.

## Background

The two-parameter Weibull distribution is widely used in reliability engineering and lifetime data analysis because of its flexibility to properly model increasing and decreasing failure rates. It has gained the interest of researchers who have worked on its various aspects, such as inference, application and parameter estimation (see Nelson 1982; Cohen 1991; Johnson et al. 1994; Meeker and Escobar 1998). Traditional parameter estimation methods call on probability plotting, least squares and maximum likelihood estimation (Lawless 1982).

A probability plotting approach is straightforward and it is best used for small size data samples. However, this estimation method has not been sufficiently accurate as reported in Mao and Li (2007). The least squares (or rank regression) method is essentially a probability plotting method that applies least squares to determine lines through points. The main disadvantage of this method is that it assigns a large weight for extreme observations, producing a large variance (Genschel and Meeker 2010).

Maximum likelihood estimation (MLE) is considered one of the most robust parameter estimation techniques. It constructs a likelihood function for a set of statistical data, which is optimized to find its extremum with respect to the distribution parameters. The MLE method can handle survival and interval data better than rank regression approaches, particularly when dealing with heavily censored data sets that contain few points of highly accurate observed data. Teimouri et al. (2013) compares the MLE method with other four methods [the Method of Logarithm Moment (MLM), the Percentile Method (PM), the L-Moments Method (LM), and the Method of Moments (MM)] to determine Weibull parameters. One of the main findings of this work is that estimation of parameters is better performed using MLE and LM estimators. However, MLE leads to likelihood equations that need to be solved numerically. Therefore, low convergence rates and efficient iterative methods must be properly addressed, which can be particularly difficult with censored data (Balakrishnan and Kateri 2008).

Recent research has been focused on obtaining new efficient numerical and statistical inference methods in order to deal with this problem. Joarder et al. (2011) consider statistical inferences of the unknown parameters of the Weibull distribution with right-censored data samples, stating that the MLE cannot lead to explicit forms of the Weibull distribution. Therefore, they propose approximate maximum likelihood estimators (AMLE), which are obtained by expanding the MLE equations in Taylor series. Also, the authors propose a fixed-point algorithm to compute the maximum likelihood estimators.

Balakrishnan and Mitra (2012) use an expectation-maximization (EM) algorithm to estimate the model parameters of the Weibull distribution of left-truncation and right-censored data. The algorithm consists of two steps: expectation step (E-step) and maximization step (M-step). The conditional expectation of the complete data likelihood is obtained with the E-step, using the incomplete observed data and current estimated value of the parameter. This expected likelihood is essentially a function of the involved parameter and its current value under which the expectation has been calculated. The expected likelihood is then maximized with respect to the parameter using the EM gradient algorithm. The E- and M-steps are then iterated until convergence. MLE and Bayes estimators are applied to calculate the survival function and the failure rate of the Weibull distribution for censored data in Guure and Ibrahim (2012). In order to estimate the survival and the failure rate functions under the MLE, the authors applied the Newton–Raphson method. Bayes estimators are obtained using a linear exponential, general entropy and squared error loss functions while a prior noninformative Bayesian approach is employed to estimate the survival function and failure rate. However, the *aposteriori* distribution function cannot be reduced to a closed form because it involves a ratio of complicated integrals. More work concerning Weibull parameter estimation can be found in Jabeen et al. (2013), Yang and Scott (2013), Guure and Ibrahim (2014), Mohammed Ahmed (2014) and Wang and Ye (2015).

Most parameter estimation methods presented in the literature are useful tools for solving practical problems, showing that the Weibull parameter estimation problem continues to be important in the research field of data analysis. Hence, it is clear that the development of general and new methods for a wider range of applications is desirable.

In this paper, an approximate analytical method to estimate the $\beta$ Weibull parameter for complete and right-censored data is proposed using perturbation theory. The method involves a systematic construction of an analytical solution to the likelihood equation for $\beta$, taking advantage of the presence of a small parameter. The solution is developed as a power series with respect to this parameter. As a result, the likelihood equation for $\beta$ is replaced by a set of simple solvable algebraic equations. These equations are explicitly solved one by one in order to obtain an increasingly accurate approximation to the true solution.

## Problem statement

Let $t_{k}|k=\overline{1,N}$, where $0<t_{1} \le t_{2} \le \cdots \le t_{N}$, be a set of lifetime data, collected from *N* products or components, consisting of *n* observed ages of failed components and $N-n$ ages of surviving components, i.e., the so-called right-censored data. Let also $\delta _{k}=1$ if $t_{k}$ is the age of a failed component and $\delta _{k}=0$ if $t_{k}$ is the age of a surviving component, so the number of failed components is:

1 $$\begin{aligned} n=\sum \limits _{k=1}^{N} \delta _{k}. \end{aligned}$$

Let us assume that the lifetime data set follows the Weibull distribution $W(\alpha ,\beta )$ with a probability density function

2 $$\begin{aligned} f\left( t|\alpha ,\beta \right) = \frac{\beta t^{\beta -1}}{\alpha ^{\beta }} \, e^{-\left( \frac{t}{\alpha }\right) ^{\beta }} \end{aligned}$$

where $\alpha$ and $\beta$ are the scale and shape parameters, respectively.

Therefore, the corresponding log-likelihood function can be recast as:

3 $$\begin{aligned} L(\alpha ,\beta ) = \left( \frac{\theta (\beta )}{\alpha } \right) ^\beta +\beta \ln \frac{\alpha }{\tau }-\ln \beta \end{aligned}$$

where

$$\begin{aligned} \ln \tau = \frac{1}{n} \sum _{k=1}^{N}\delta _{k}\ln t_{k} \qquad \text {and} \qquad \theta (\beta ) = \left( \frac{1}{n} \sum _{k=1}^{N} t_{k}^\beta \right) ^{1/\beta }. \end{aligned}$$

The MLE method states that the most probable values of $\alpha$ and $\beta$ correspond to the extremum of (3), or equivalently to the existence and uniqueness of the solution $(\alpha ^{*},\beta ^{*})$ of the following system equations:

4 $$\begin{aligned} \left\{ \begin{array}{l} \frac{\partial L(\alpha , \beta )}{\partial \alpha } =0 \\ \frac{\partial L(\alpha , \beta )}{\partial \beta } = 0. \end{array} \right. \end{aligned}$$

The system of Eq. (4) can be expressed as:

5 $$\begin{aligned}&\alpha =\theta (\beta ), \end{aligned}$$

6 $$\begin{aligned}&\frac{1}{\beta _1}-\frac{1}{\beta } -\frac{ \sum \nolimits _{k=1}^{N} \left( \ln \frac{t_{N}}{t_{k}} \right) t_{k}^{\beta }}{ \sum \nolimits _{k=1}^{N} t_{k}^{\beta }}=0 \end{aligned}$$

where

7 $$\begin{aligned} \beta _{1} = \left( \frac{1}{n}\sum \limits _{k=1}^{N}\delta _{k} \ln \frac{t_{N}}{t_{k}} \right) ^{-1}. \end{aligned}$$

$\beta ^{*}$ is then estimated solving Eq. (6). Equation (5) is the closed-form expression for the MLE of $\alpha$. Thus, $\alpha ^{*}$ can be calculated by substituting the estimated value of $\beta ^{*}$ into Eq. (5). $\beta$ is usually solved for employing numerical or graphical methods, which involve inaccuracies and numerical problems. So, the main aim of this research is to obtain an analytical solution to Eq. (6), allowing the shape parameter $\beta$ to be explicitly found.

## Existence and uniqueness of the likelihood estimate

The existence and uniqueness of the solution of the MLE equation have already been proved in Balakrishnan and Kateri (2008) and Farnum and Booth (1997) using Cauchy-Schwarz inequality. A different proof is presented here, leading to our proposed analytical solution for the $\beta$ parameter.

Let us denote:

8 $$\begin{aligned} x_{k}& = {} \left( \frac{t_{k}}{t_{N}}\right) ^{\beta _{1}},\qquad k=\overline{1,N}, \\ \zeta& = {} \frac{\beta }{\beta _{1}}, \end{aligned}$$

where $0<x_{k}\le 1$ and $x_{N}=1$. Hence, it can be seen that

9 $$\begin{aligned} Z(\zeta )\equiv 1-\frac{1}{\zeta } +r(\zeta )=0, \end{aligned}$$

after multiplying Eq. (6) by $\beta _{1}$. Here,

10 $$\begin{aligned} r(\zeta )\equiv \frac{ \sum \nolimits _{k=1}^{N} \left( \ln x_{k} \right) x_{k}^{\zeta }}{ \sum \nolimits _{k=1}^{N} x_{k}^{\zeta }}. \end{aligned}$$

In order to prove the existence and uniqueness of the solution of Eq. (9), the global monotonicity of $Z(\zeta )$ and its asymptotic behavior, in the limits $\zeta \rightarrow 0+$ and $\zeta \rightarrow +\infty$, is developed. Let us consider that $0<x_{1}\le x_{2}\le \cdots \le x_{N}=1$, where $N\ge 2$, is a monotonous nondecreasing data sequence. Also, let us suppose that there must be at least two different statistical data sets, i.e.,

11 $$\begin{aligned} \exists \, m\in \{\overline{1,N-1}\}: x_{m}<x_{N}=1 \quad {\mathrm{and}} \quad x_{m+1}=x_{N}=1. \end{aligned}$$

It can be proved that $r(\zeta )$ is continuous and monotonously increasing on $\zeta >0$, since the derivative of $r(\zeta )$ is positive for all $\zeta >0$:

$$\begin{aligned} \frac{dr(\zeta )}{d\zeta }& = {} \frac{ \left( \sum \nolimits _{k=1}^{N}(\ln x_{k})^2 x_{k}^{\zeta } \right) \left( \sum \nolimits _{k=1}^{N} x_{k}^{\zeta } \right) - \left( \sum \nolimits _{k=1}^{N} (\ln x_{k}) x_{k}^{\zeta } \right) ^{2}}{ \left( \sum \nolimits _{k=1}^{N} x_{k}^{\zeta } \right) ^2 }\\& = {} \frac{ \sum \nolimits _{x_{i}>x_{j}} \left( \ln \frac{x_{i}}{x_{j}} \right) ^{2} x_{i}^{\zeta } x_{j}^{\zeta }}{ 2 \left( \sum \nolimits _{k=1}^{N} x_{k}^{\zeta } \right) ^2 } >0. \end{aligned}$$

This implies that $Z(\zeta )$ is also continuous and monotonously increasing on $\zeta >0$. On the other hand, $r(\zeta )$ is bounded and its boundaries can be obtained from the asymptotic behavior of $r(\zeta )$ in the limits $\zeta \rightarrow 0+$ and $\zeta \rightarrow +\infty$. In the limit $\zeta \rightarrow 0+$, the asymptotic series expansions of $\zeta$ is given by:

$$\begin{aligned} \sum \limits _{k=1}^{N} x_{k}^{\zeta }=\sum \limits _{k=1}^{N} e^{\zeta \ln x_{k}} = \sum \limits _{k=1}^{N} \left( 1+{\mathcal{O}}(\zeta ) \right) = N\left( 1+{\mathcal{O}}(\zeta ) \right) \end{aligned}$$

and

$$\begin{aligned} \sum \limits _{k=1}^{N} \left( \ln x_{k} \right) x_{k}^{\zeta } = \sum \limits _{k=1}^{N} \left( \ln x_{k} \right) \left( 1+{\mathcal{O}}(\zeta ) \right) \end{aligned}$$

where ${\mathcal{O}}(\zeta )$ is the big Landau O notation.

Substitution of these results into (10) yields the following asymptotic equation for $r(\zeta )$ in the limit $\zeta \rightarrow 0+$:

12 $$\begin{aligned} r(\zeta ) = \frac{1}{N}\sum \limits _{k=1}^{N} \ln x_{k}+{\mathcal{O}}(\zeta ) \end{aligned}$$

which states that $r(\zeta )\rightarrow \frac{1}{N}\sum \nolimits _{k=1}^{N}\ln x_{k}$ as $\zeta \rightarrow 0+$.

Now, it can be shown that $r(\zeta )\rightarrow 0$ as $\zeta \rightarrow +\infty$. Hence,

$$\begin{aligned} \sum _{k=1}^{N} x_k^{\zeta }=x_1^{\zeta }+x_2^{\zeta }+\cdots +x_m^{\zeta }+(N-m)= (N-m) \Bigl \{ 1+{\mathcal{O}} \left( x_{m}^{\zeta } \right) \Bigr \} \end{aligned}$$

due to (11) and noticing that $x_{k}=x_{N}=1$ for all $m<k\le N$ and $0<x_{m}<1$, $x_m^{\zeta }\rightarrow 0$ as $\zeta \rightarrow +\infty$. Therefore, Eq. (10) and this last result, along with the fact that $\ln x_{k}=0$ for $k>m$, show that the asymptotic expansion for $r(\zeta )$ in the limit $\zeta \rightarrow +\infty$ is given by:

13 $$\begin{aligned} r(\zeta ) = \frac{ \sum \nolimits _{k=1}^m \left( \ln x_{k} \right) x_k^{\zeta } }{ (N-m) \Bigl \{ 1+{\mathcal{O}} \left( x_{m}^{\zeta } \right) \Bigr \} } = \frac{1}{N-m}\sum \limits _{k=1}^m \left( \ln x_{k} \right) x_{k}^{\zeta } \Bigl \{ 1+{\mathcal{O}} \left( x_{m}^{\zeta } \right) \Bigr \} = {\mathcal{O}} \left( x_{m}^{\zeta } \right) , \end{aligned}$$

i.e., $$r(\zeta )\rightarrow 0$$ as $$\zeta \rightarrow +\infty$$.

It follows from the asymptotic equations (12) and (13) that $Z(\zeta )$ is a continuous and monotonously increasing function for $\zeta >0$ with the following asymptotic behavior:

14 $$\begin{aligned} Z(\zeta ) = 1-\frac{1}{\zeta }+\frac{1}{N}\sum \limits _{k=1}^{N} \ln x_{k}+{\mathcal{O}}(\zeta )\rightarrow -\infty \quad {\mathrm{as}}\quad \zeta \rightarrow 0+, \end{aligned}$$

15 $$\begin{aligned} Z(\zeta )=1-\frac{1}{\zeta }+{\mathcal{O}} \left( x_{m}^{\zeta } \right) \rightarrow 1 \quad {\mathrm{as}}\quad \zeta \rightarrow +\infty . \end{aligned}$$

As a result, there exists a unique value $\zeta ^*$ such that $Z(\zeta ^*)=0$ since (14) and (15).

Let us determine the solution interval of $\zeta ^{*}$. It follows from Eq. (9) that $0=Z(\zeta ^*)<1-{\zeta ^*}^{-1}$ since $r(\zeta )<0$. As a result $\zeta ^*>1$. On the other hand, it can be seen that $r(1)<r(\zeta ^*)=-1+{\zeta ^*}^{-1}$ for $\zeta ^*>1$, i.e., ${\zeta ^*}^{-1}>1+r(1)$ since $r(\zeta )$ is monotonously increasing. At the same time, ${\zeta ^*}^{-1}>0$. Therefore, ${\zeta ^*}^{-1}>\max \left\{ 1+r(1),0\right\}$, where

16 $$\begin{aligned} r(1)=\frac{ \sum \nolimits _{k=1}^{N} \left( \ln x_{k} \right) x_{k}}{ \sum \nolimits _{k=1}^{N} x_{k}}. \end{aligned}$$

Summarizing, it can be concluded that

17 $$\begin{aligned} 1+r(1)<\frac{1}{\zeta ^{*}}<1. \end{aligned}$$

**Fig. 1:**
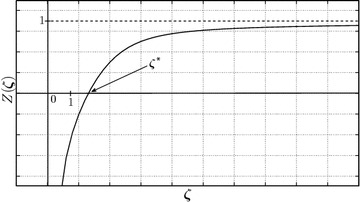
Plot of the $Z(\zeta )$ function

The asymptotical behavior of $Z(\zeta )$ (in the limits $\zeta \rightarrow 0+$ and $\zeta \rightarrow +\infty$) and $\zeta ^{*}$ are illustrated in Fig. 1. However, the expression $1+r(1)$ can be negative for some statistical data sets. In this case, the boundary estimation for $\zeta ^{*}$ becomes:

18 $$\begin{aligned} 1<\zeta ^{*}<\infty . \end{aligned}$$

It can be observed that this interval is too large. Then, a better estimation of the right boundary is required. For this, let us denote:

19 $$\begin{aligned} \zeta =1+z, \end{aligned}$$

where $z>0$ in virtue of (17). Then, Eq. (9) can be written as:

20 $$\begin{aligned} (1+z)\sum \limits _{k=1}^{N} (\ln x_{k})x_{k}^{1+z} + z \sum \limits _{k=1}^{N} x_{k}^{1+z}=0. \end{aligned}$$

The solution $\beta ^{*}$ is found from Eq. (20), which can be substituted into $\beta ^{*}=\beta _{1}\,\zeta ^{*}=\beta _{1} (1+z)$ according to Eqs. (8) and (19). This way, $\alpha ^{*}$ can be calculated by substituting the estimated $\beta ^{*}$ value into Eq. (5).

## A perturbative approach to estimate the shape parameter

Perturbation theory is employed in this section to solve Eq. (20). It allows the representation of $\zeta ^{*}$ to be asymptotically expanded, which in turn can be conveniently truncated to obtain an analytical solution to Eq. (20).

After expanding each term of Eq. (20) in Taylor series and dividing the entire equation by the expression $\sum \nolimits _{k=1}^{N}x_{k}\left[ (\ln x_{k})^{2}+\ln x_{k}+1\right]$, it can be written as:

21 $$\begin{aligned} \sum \limits _{m=0}^{\infty }\frac{z^{m}}{m!}\sigma _{m}=0 \end{aligned}$$

where

22 $$\begin{aligned} \sigma _{m}=\frac{ \sum \nolimits _{k=1}^{N}x_{k}(\ln x_{k})^{m-1} \left[ (\ln x_{k})^{2}+m \ln x_{k}+m \right] }{ \sum \nolimits _{k=1}^{N}x_{k} \left[ (\ln x_{k})^{2}+\ln x_{k}+1 \right] }. \end{aligned}$$

The simplest analytical solution to Eq. (21) can be found by truncating the power series in (21) at $m=1$. As a result, the following expression can be obtained:

$$\begin{aligned} \sigma _{0}+\sigma _{1} z\approx 0. \end{aligned}$$

The solution to this equation is given by:

$$\begin{aligned} z\approx -\frac{\sigma _{0}}{\sigma _{1}} = -\sigma _{0}, \end{aligned}$$

and subsequently denote

23 $$\begin{aligned} \varepsilon \equiv -\sigma _{0}, \end{aligned}$$

where $0<-\sigma _{0}<1$ (see “Appendix”). Therefore, the exact solution to Eq. (21) can be expanded in a power series with respect to $\varepsilon$, so that a solution to *z* can be written as:

24 $$\begin{aligned} z= \varepsilon \xi _{1}+\varepsilon ^{2}\xi _{2}+\varepsilon ^{3}\xi _{3}+\cdots = \sum \limits _{q=1}^{\infty }\varepsilon ^{q}\xi _{q}. \end{aligned}$$

Noticing that $z^{m}$ can be recast as:

25 $$\begin{aligned} z^{m} = \sum \limits _{q_{1} \cdots q_{m}=1}^{\infty } \varepsilon ^{\sum \limits _{k={1}}^{m}q_{k}} \left( \prod \limits _{j=1}^{m}\xi _{q_{j}} \right) = \sum \limits _{Q=m}^{\infty } \varepsilon ^{Q} \mathop {\mathop {\sum }\limits _{q_{1}\cdots q_{m}}}\limits _{\sum \limits _{k=1}^{m}q_{k}=Q} \left( \prod \limits _{j=1}^{m}\xi _{q_{j}} \right) \end{aligned}$$

where $\mathop {\mathop {\sum }\nolimits _{q_{1}\cdots q_{m}}}\nolimits _{\sum \nolimits _{k=1}^{m}q_{k}=Q}$ means summation over all $q_{1}, q_{2},\ldots,q_{m}$ such that $\sum \nolimits _{k=1}^{m}q_{k}=Q$. Substitution of (25) into (21), and taking on account of (23), Eq. (21) has the following expression:

26 $$\begin{aligned}& \sigma _{0}+ \sum \limits _{Q=1}^{\infty }\varepsilon ^{Q} \sum \limits _{m=1}^{Q}\frac{\sigma _{m}}{m!} \mathop {\mathop {\sum }\limits _{q_{1}\cdots q_{m}}}\limits _{\sum \limits _{k=1}^{m}q_{k}=Q} \left( \prod \limits _{j=1}^{m}\xi _{q_{j}} \right)& \\& \quad =\varepsilon (-1+\sigma _{1}\xi _{1})+ \sum \limits _{Q=2}^{\infty }\varepsilon ^{Q} \sum \limits _{m=1}^{Q}\frac{\sigma _{m}}{m!} \mathop {\mathop {\sum }\limits _{q_{1}\cdots q_{m}}}\limits _{\sum \limits _{k=1}^{m}q_{k}=Q} \left( \prod \limits _{j=1}^{m}\xi _{q_{j}} \right) =0.&\end{aligned}$$

Each coefficient of the power series in the left-hand-side of Eq. (26) is zero according to the perturbation theory. As a result, the following infinite system of algebraic equations can be obtained:

27 $$\begin{aligned} \left\{ \begin{array}{l} -1+\sigma _{1}\xi _{1}=0,\\[12pt] \sum \nolimits _{m=1}^{Q}\frac{\sigma _{m}}{m!} \mathop {\mathop {\sum }\nolimits _{q_{1} \cdots q_{m}}}\nolimits _{\sum \nolimits _{k=1}^{m}q_{k}=Q} \left( \prod \nolimits _{j=1}^{m}\xi _{q_{j}} \right) =0, \qquad Q=2,3,\ldots \end{array} \right. \end{aligned}$$

All coefficients of the asymptotic series (24) can be determined by iteratively solving the system of equations (27). Thus, the complete asymptotic series (24) is fully established. Generally, higher-order terms in the series (24) become successively smaller for $\varepsilon$ small. Therefore a good approximation is obtained when the power series is truncated using a few terms. For example, if series (27) is truncated at term $Q=4$, the system becomes:

28 $$\begin{aligned} \left\{ \begin{array}{l} \sigma _{1}\xi _{1}-1=0, \\[12pt] \sigma _{1}\xi _{2}+ \frac{\sigma _{2}}{2}\xi _{1}^{2}=0, \\[12pt] \sigma _{1}\xi _{3}+ \frac{\sigma _{2}}{2}(2\xi _{1}\xi _{2})+ \frac{\sigma _{3}}{3!}\xi _{1}^{3}=0, \\[12pt] \sigma _{1}\xi _{4}+ \frac{\sigma _{2}}{2}(2\xi _{1}\xi _{3}+\xi _{2}^{2})+ \frac{\sigma _{3}}{3!}(3\xi _{1}^{2}\xi _{2})+ \frac{\sigma _{4}}{4!}\xi _{1}^{4}=0. \end{array} \right. \end{aligned}$$

A consistent solution to (28) can be obtained by successively solving each of its equations:

29 $$\begin{aligned} \begin{array}{c} \xi _{1}= 1, \qquad \xi _{2}=- \frac{\sigma _{2}}{2}, \qquad \xi _{3}= \frac{\sigma _{2}^{2}}{2} - \frac{\sigma _{3}}{6}, \\[12pt] \xi _{4}=- \frac{5\sigma _{2}^{3}}{8} + \frac{5\sigma _{3}\sigma _{2}}{12} - \frac{\sigma _{4}}{24}. \end{array} \end{aligned}$$

Hence, substitution of these coefficients into Eq. (24) yields the asymptotic solution to Eq. (26):

30 $$\begin{aligned} z& = {} -\sigma _{0}-\frac{\sigma _{2}}{2} \left( \sigma _{0} \right) ^{2}+ \left( \frac{\sigma _{3}}{6} - \frac{\sigma _{2}^{2}}{2} \right) \left( \sigma _{0} \right) ^{3} \\[12pt]&\quad + \left( \frac{5\sigma _{3}\sigma _{2}}{12} - \frac{5\sigma _{2}^{3}}{8} - \frac{\sigma _{4}}{24} \right) \left( \sigma _{0} \right) ^{4} +{\mathcal{O}} \biggl ( (\sigma _{0})^{5} \biggr ). \end{aligned}$$

The asymptotic solution to $\zeta ^{*}$ in Eq. (9) is obtained by substituting (30) into (19):

31 $$\begin{aligned} \zeta ^{*}& = {} 1-\sigma _{0}-\frac{\sigma _{2}}{2} \left( \sigma _{0} \right) ^{2}+ \left( \frac{\sigma _{3}}{6} - \frac{\sigma _{2}^{2}}{2} \right) \left( \sigma _{0} \right) ^{3} \\[12pt]&\quad + \left( \frac{5\sigma _{3}\sigma _{2}}{12} - \frac{5\sigma _{2}^{3}}{8} - \frac{\sigma _{4}}{24} \right) \left( \sigma _{0} \right) ^{4} +{\mathcal{O}} \biggl ( (\sigma _{0})^{5} \biggr ). \end{aligned}$$

Finally, it can be seen from (8) that $\beta =\beta _{1}\cdot \zeta$. The approximate analytical solution for $\beta ^{*}$ is then obtained as:

32 $$\begin{aligned} \beta ^{*} = \beta _{1}\cdot \zeta ^{*}& = {} \beta _{1} \left\{ 1-\sigma _{0}-\frac{\sigma _{2}}{2} \left( \sigma _{0} \right) ^{2}+ \left( \frac{\sigma _{3}}{6} - \frac{\sigma _{2}^{2}}{2} \right) \left( \sigma _{0} \right) ^{3} \right. \\[12pt]&\quad + \left. \left( \frac{5\sigma _{3}\sigma _{2}}{12} - \frac{5\sigma _{2}^{3}}{8} - \frac{\sigma _{4}}{24} \right) \left( \sigma _{0} \right) ^{4} +{\mathcal{O}} \biggl ( (\sigma _{0})^{5} \biggr ) \right\} . \end{aligned}$$

In turn, the scale parameter $\alpha ^{*}$ can be determined by substituting the result of (32) into Eq. (5).

## Cases of study

Three study cases are shown in this section to illustrate the application of our proposed analytical method for the estimation of the Weibull $\beta$ parameter. The first study considers right-censored data set found in Balakrishnan and Kateri (2008), where a graphical solution for the determination of the MLE shape parameter is employed. In a second study, the proposed method is applied to right-censored data used in Balakrishnan and Mitra (2012). Finally, sets of lifetime data are randomly generated combining different censoring rates and sample sizes, in order to cover a wider range of data sampling scenarios that might be encountered in practical applications. Corresponding Weibull parameters for each data set are accordingly estimated.

For the first two cases, the $\beta$ parameter was also estimated using a Newton-Rapshon algorithm with the purpose of illustrating the advantage of our proposed analytical method, where $\beta$ is obtained by a single equation.

### Case 1

The censored data set provided by Dodson (2006) and analyzed by Balakrishnan and Kateri (2008) is shown in Table 1. It provides twenty identical grinders which were tested with a ending time $t=152.7$. Twelve grinders failed in this period of time. Values of $\sigma _m$, $m=\overline{0,4}$, are obtained from Eq. (22): $\sigma _0=-0.2244$, $\sigma _1=1.0$, $\sigma _2=-0.3397$, $\sigma _3=0.3617$, $\sigma _4=-0.3320$. The Weibull $\beta$ parameter is estimated from Eq. (32) and these values of $\sigma _m$. The result is substituted into Eq. (5) to obtain $\alpha$. Results are presented in Table 2 along with the parameters estimated by a graphical method in Balakrishnan and Kateri (2008).

**Table 1:** Lifetime data for Case 1

*k*	$t_k$	$\delta _k$	*k*	$t_k$	$\delta _k$	*k*	$t_k$	$\delta _k$	*k*	$t_k$	$\delta _k$
1	12.5	1	6	95.5	1	11	125.6	1	16	152.7	0
2	24.4	1	7	96.6	1	12	152.7	1	17	152.7	0
3	58.2	1	8	97.0	1	13	152.7	0	18	152.7	0
4	68.0	1	9	114.2	1	14	152.7	0	19	152.7	0
5	69.1	1	10	123.2	1	15	152.7	0	20	152.7	0

**Table 2:** Parameter estimates for Case 1

Parameters	Approximate analytical method	Approach in Balakrishnan and Kateri (2008)	NR method $\beta _{0}=2$
$\beta$	1.6466	1.647	1.6466 (6 iterations)
$\alpha$	162.22306	162.223	162.2330

The MLE was combined with the Newton–Raphson method using a convergence tolerance set to 0.001 and an initial guess $\beta _{0}=2$, which corresponds to our $\beta$ solution when it is rounded up to the next integer. This last criterion is adopted from the well-known fact that an appropriate initial value (close to the desired solution) ensures the convergence of the NR method within few iterations.

It can be observed from Table 2 that the parameters obtained from our analytical method match closely the estimates of the NR method and graphical method proposed in Balakrishnan and Kateri (2008). Therefore, it can be stated that our proposed parameter estimation methodology not only works well for this case, but it also directly provides $\beta$ from an explicit expression [Eq. (32)].

### Case 2

In this case, the data set is provided by Balakrishnan and Mitra (2012) and reproduced in Table 3. It is used to assess again the efficacy of our proposed parameter estimation method. This data set was numerically produced using the Weibull distribution with parameter values $\alpha _{\mathrm{true}}=35$ and $\beta _{\mathrm{true}}=3$. It can be considered as lifetime data of power transformers in the electrical industry, with information of installation and failure dates. The data considers units that failed before year 2008, which was set as the year of censoring.

**Table 3:** Simulated data set provided by Balakrishnan and Mitra (2012)

Simulated data set provided by Balakrishnan and Mitra (2012) No.	Installation year	Failure year	No.	Installation year	Failure year	No.	Installation year	Failure year	No.	Installation year	Failure year
1	1984	–	26	1986	–	51	1982	–	76	1974	2006
2	1990	2001	27	1987	–	52	1981	–	77	1978	1995
3	1983	2002	28	1990	1997	53	1986	–	78	1962	1993
4	1981	2000	29	1980	1996	54	1980	1990	79	1963	–
5	1985	–	30	1980	–	55	1980	1994	80	1960	1998
6	1991	–	31	1981	–	56	1982	–	81	1962	2007
7	1982	–	32	1983	1997	57	1990	2008	82	1960	1990
8	1990	–	33	1980	–	58	1985	–	83	1962	1980
9	1983	1999	34	1984	–	59	1983	–	84	1961	1981
10	1992	–	35	1982	–	60	1982	–	85	1964	1989
11	1983	–	36	1980	–	61	1963	1996	86	1964	1987
12	1989	–	37	1985	2007	62	1963	2001	87	1960	2006
13	1985	–	38	1993	–	63	1961	1998	88	1961	1992
14	1982	–	39	1983	–	64	1961	1992	89	1964	–
15	1983	–	40	1980	–	65	1960	1984	90	1963	1991
16	1981	–	41	1981	2001	66	1964	2004	91	1973	–
17	1985	–	42	1989	–	67	1961	1994	92	1964	–
18	1981	–	43	1993	–	68	1977	1998	93	1972	1984
19	1988	2002	44	1983	–	69	1963	1987	94	1962	2007
20	1983	–	45	1993	–	70	1960	1991	95	1963	1997
21	1984	–	46	1987	–	71	1961	1983	96	1964	1987
22	1989	–	47	1994	–	72	1964	1995	97	1964	2002
23	1988	–	48	1985	2007	73	1963	1998	98	1971	–
24	1982	–	49	1981	–	74	1961	2001	99	1965	1990
25	1981	–	50	1983	2004	75	1960	1988	100	1962	1994

The parameter values obtained for this case are presented in Table 4. Similarly to Case 1, selection for the initial value of the NR method is the round up integer of the $\beta$ solution obtained using our analytical method, with a tolerance of 0.001.

**Table 4:** Parameter estimates for Case 2

Parameters	Approximate analytical method	NR method $\beta _{0}=4$
$\beta$	3.205	3.2506 (8 iterations)
$\alpha$	35.245	35.2084

It can be observed again that the parameters obtained with our analytical method match quite well the estimates of the NR program. This case is another example that shows the efficacy of our proposed analytical method for the determination of Weibull parameters.

### Case 3

The simulation approach of Zhou et al. (2013) was adopted to generate different sets of lifetime data at prespecified points of time. The generated data mimic a time-censored sampling scenario for a hypothetical number of transformer units, which operate at the same time. In addition, it is assumed that a population of units is homogenous with a fixed censoring time *C*, and each individual unit has a lifetime $t_k$, $k=\overline{1,M}$, where *M* denotes the total number of units. Each $t_k$ is identically considered an independent random variable that follows a specific probability distribution. Finally, lifetime data is characterized by the censoring rate *CR*, defined as the proportion of censored data and calculated as the number of suspensions divided by the sample size.

The sample sizes employed in this study were 10, 20, 50, 100, 500 and 1000. Censoring rates were fixed at 0, 20 and $80\,\%$. Each group of simulated lifetime data required *M* numbers of $t_k$ that were randomly generated from a two-parameter Weibull distribution with prespecified values of $\alpha _{\mathrm{true}}=3.0$ and $\beta _{\mathrm{true}}=1.5$. Censoring times were chosen to have a common value, which is calculated as $F_x^{-1} (p;\alpha ,\beta )$, where *p* is the probability of a unit, starting at time 0, fails before reaching censoring time *C*. *p* was fixed for each *CR* at 1.0, 0.8 and 0.2. Then, a lifetime data set is generated through the comparison of lifetime units and a selected censoring time: If $t_{k}$ is less than or equal to *C*, the unit is failed. Otherwise, the unit is in suspension with lifetime data censored at time *C*.

This study was specially designed to bring about the effectiveness of our proposed analytical MLE method for Weibull parameters. Our proposed method was also compared in this work with the L-Moments estimation method presented in Teimouri et al. (2013), which is based on linear combination of order statistics and provides closed-form expressions for Weibull parameters.

Weibull parameters were obtained for each simulated data set using our analytical MLE method and the L-Moments method (see Table 5). It can be observed from these results that both methods provide a close match to $\alpha _{\mathrm{true}}$ and $\beta _{\mathrm{true}}$. Our analytical MLE method provides estimates that are closer to $\alpha _{\mathrm{true}}$ and $\beta _{\mathrm{true}}$ for censored data sets. Therefore, we can state from these results that the L-Moments method is effective for complete data sets, whereas our analytical MLE method is effective for complete data sets as well as right-censored data sets.

**Table 5:** Case 3. Parameter estimates for different simulated data sets

*CR* (%)	*M*	L-Moments method		Approximate analytical method	
		$\alpha$	$\beta$	$\alpha$	$\beta$
0	10	3.0522	2.1521	3.0563	2.5286
	20	3.4026	1.3887	3.4289	1.4851
	50	2.7332	1.3915	2.7112	1.3497
	100	2.9970	1.5129	2.9860	1.4877
	500	3.1178	1.5677	3.0670	1.4772
	1000	3.0152	1.5289	2.9624	1.4342
20	10	2.6830	1.9116	2.9353	1.7227
	20	2.9035	1.8576	3.3553	1.4175
	50	2.5856	1.8822	2.8145	1.5906
	100	2.8119	2.1616	2.9557	1.9031
	500	2.7591	1.8930	3.0847	1.4296
	1000	2.7483	1.9360	3.0350	1.5349
80	10	1.0279	4.5619	1.4874	2.1709
	20	1.0871	12.9564	2.4157	2.2982
	50	1.0709	7.8137	3.0147	1.4884
	100	1.0553	6.1198	3.4455	1.2138
	500	1.0757	9.3024	2.8698	1.6711
	1000	1.0709	8.2974	2.9130	1.5363

An additional analysis was carried out to compare both methods in terms of the mean-squared error and bias. For this purpose, the bias and the mean-squared error were computed for data sets of size $M=10,20,\ldots ,1000$, which were randomly generated from a two-parameter Weibull distribution with prespecified values of $(\alpha _{\mathrm{true}},\beta _{\mathrm{true}})=(0.5,0.5), (1.0,1.0),(3.0, 1.5)$, and the number of simulations for each data set was 100. The mean-squared error and bias are defined as follows (Teimouri et al. 2013):

$$\begin{aligned} {\mathrm{Bias}}(\alpha ^{*})& = {} \frac{1}{\eta }\sum \limits _{i=1}^{\eta } (\alpha ^{*}_{i}-\alpha _{\mathrm{true}}),\\ {\mathrm{Bias}}(\beta ^{*})& = {} \frac{1}{\eta }\sum \limits _{i=1}^{\eta } (\beta ^{*}_{i}-\beta _{\mathrm{true}}), \\ {\mathrm{MSE}}(\alpha ^{*})& = {} \frac{1}{\eta }\sum \limits _{i=1}^{\eta } (\alpha ^{*}_{i}-\alpha _{\mathrm{true}})^{2} \end{aligned}$$

and

$$\begin{aligned} {\mathrm{MSE}}(\beta ^{*}) = \frac{1}{\eta }\sum \limits _{i=1}^{\eta } (\beta ^{*}_{i}-\beta _{\mathrm{true}})^{2}, \end{aligned}$$

where $\eta$ is the number of simulations and $\alpha ^{*}_{i}$ and $\beta ^{*}_{i}$ are estimators of $\alpha$ and $\beta$ for the *i*-th simulation, respectively.

**Fig. 2:**
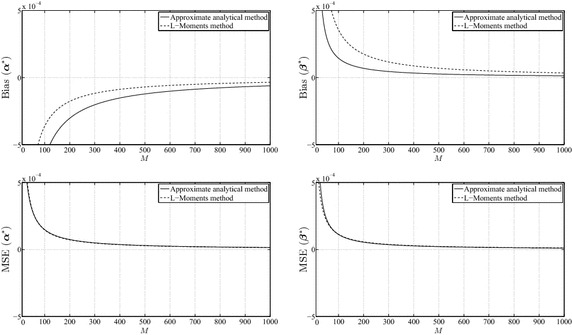
Biases of $\alpha ^{*}$, $\beta ^{*}$ and mean-squared errors of $\alpha ^{*}$, $\beta ^{*}$ for $\alpha_{\mathrm{true}} =0.5$ and $\beta_{\mathrm{true}} =0.5$

**Fig. 3:**
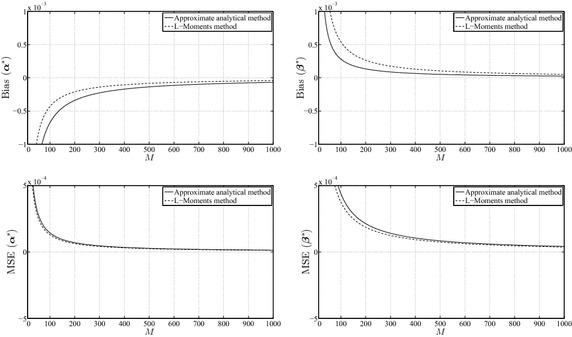
Biases of $\alpha ^{*}$, $\beta ^{*}$ and mean-squared errors of $\alpha ^{*}$, $\beta ^{*}$ for $\alpha_{\mathrm{true}} =1.0$ and $\beta_{\mathrm{true}} =1.0$

**Fig. 4:**
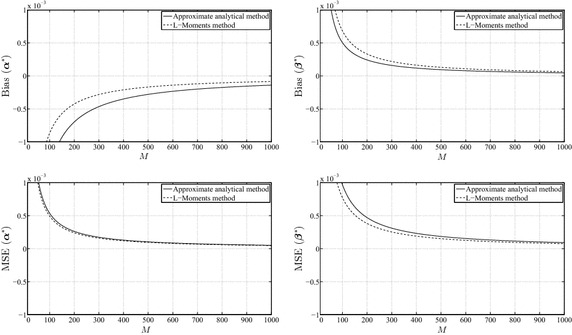
Biases of $\alpha ^{*}$, $\beta ^{*}$ and mean-squared errors of $\alpha ^{*}$, $\beta ^{*}$ for $\alpha_{\mathrm{true}} =3.0$ and $\beta_{\mathrm{true}} =1.5$

Figures 2, 3 and 4 illustrate the behavior of the mean-squared error and bias for $\alpha ^{*}$ and $\beta ^{*}$. It can be concluded from these results that the L-Moments method has more bias in the estimates of $\beta ^{*}$, whereas our analytical MLE method has more bias in the estimates of $\alpha ^{*}$. However, the bias and the mean-squared error in both methods tend to consistently decrease with the increase of the size of the data sets. The difference between both methods, with respect to the mean-squared error criterion, is very small, mainly for large *M* where the difference is practically indistinguishable. In addition, the amount of bias and mean-squared error behaves in a consistent manner over different values of the parameters.

## Conclusions

An analytical approach is developed in this work to estimate the Weibull parameter $\beta$ for complete and right-censored data using perturbation theory. The idea behind this method is to formally expand the $\beta$ solution to its likelihood equation around point 1.0 as a power series in $\varepsilon$, which turns out to be a small parameter. In fact, if $\varepsilon$ is zero, the equation is exactly solvable. Therefore, the problem is reduced to find the asymptotic behavior of the best approximation to the true solution within $\varepsilon ,\varepsilon ^{2},\ldots$ Thus, perturbation theory leads to an expression for the desired solution in terms of a formal power series in a “small” parameter that quantifies the deviation from the exactly solvable problem. Hence, an approximate analytical solution for $\beta$ parameter is obtained by truncating the series at a prespecified order.

Our analytical method for estimation of the Weibull parameter was tested on several lifetime data sets. This way, it was concluded that the performance of our proposed method was satisfactory for all lifetime data sets with different combinations of sample sizes with small and heavy censoring. These data sets cover a wide range of practical scenarios that our method can easily deal with.

The main conclusion that can be drawn from this work is that the use of the formulations described in “Existence and uniqueness of the likelihood estimate” and “A perturbative approach to estimate the shape parameter” sections allows the analytical obtention of $\beta$. This method was not only numerically tested using common and practical data sets, but it was also theoretically and mathematically proved. Our approach efficiently estimates $\beta$ employing a single equation, with no need of graphical or iterative procedures.

Finally, it is worth mentioning that although the estimation of Weibull parameters under right-censored scheme was considered, the proposed method can be extended to other censoring schemes such as left-truncation and hybrid. Additional work is required in this direction, which is currently considered by the authors.

## Appendix

Inequality $-1<\sigma _{0}<0$ is proved here. First see that Eq. (22) yields

33 $$\begin{aligned} \sigma _{0}=\frac{ \sum \nolimits _{k=1}^{N}x_{k}(\ln x_{k})}{ \sum \nolimits _{k=1}^{N}x_{k} \left[ (\ln x_{k})^{2}+\ln x_{k}+1 \right] }. \end{aligned}$$

Since $0<x_{k}<1$, $\ln x_{k}<0$. As a result,

$$\begin{aligned} \sum \limits _{k=1}^{N}x_{k}(\ln x_{k})<0 . \end{aligned}$$

On the other hand, $(\ln x_{k})^{2}+\ln x_{k}+1>0$ $\forall$ $x_{k}$, so

$$\begin{aligned} \sum \limits _{k=1}^{N}x_{k} \left[ (\ln x_{k})^{2}+\ln x_{k}+1 \right] >0 . \end{aligned}$$

which shows that $\sigma _{0}<0$. Therefore, we see that

34 $$\begin{aligned} \sigma _{0}=\frac{ \sum \nolimits _{k=1}^{N}x_{k}(\ln x_{k}+1)^{2}}{ \sum \nolimits _{k=1}^{N}x_{k} \left[ (\ln x_{k})^{2}+\ln x_{k}+1 \right] }-1>-1. \end{aligned}$$

Therefore $$-1<\sigma _{0}<0$$, Q.E.D.
